# Organ-Specific Small Protein Networks in 100 kDa Ultrafiltrates: Functional Analysis and Implications for Neuroregenerative Medicine

**DOI:** 10.3390/ijms26146659

**Published:** 2025-07-11

**Authors:** Jakub Peter Slivka, Chris Bauer, Tasneem Halhouli, Alexander Younsi, Michelle B. F. Wong, Mike K. S. Chan, Thomas Skutella

**Affiliations:** 1 Reviva, Plzenska 47, 252 19 Chrastany, Czech Republic; jakub.slivka@reviva.info; 2 MicroDiscovery, 10405 Berlin, Germany; 3 Institute for Anatomy and Cell Biology, Medical Faculty, University of Heidelberg, Im Neuenheimer Feld 307, 69120 Heidelberg, Germany; 4 Department of Neurosurgery, Heidelberg University Hospital, 69120 Heidelberg, Germany; 5 Stellar Biomolecular Research GmbH, Klosterstrasse 205a, 67480 Edenkoben, Germany; 6 EW European Wellness International GmbH, Sommerhalde 21, 72184 Eutingen, Germany

**Keywords:** small proteins, ultrafiltration, proteomics, organ specificity

## Abstract

In this research, the proteomic landscape of 100 kDa protein extract sourced from rabbit brain was compared to extracts from liver and from organ mixture (OM). Our aim was to compare the efficacy of Nanomised Organo Peptides (NOP) ultrafiltrates from two different tissues and a tissue mixture for inducing neurite outgrowth, and subsequently to identify the molecular networks and proteins that could explain such effects. Proteins were isolated by gentle homogenization followed by crossflow ultrafiltration. Proteomic evaluation involved gel electrophoresis, complemented by mass spectrometry and bioinformatics. GO (Gene Ontology) and protein analysis of the mass spectrometry results identified a diverse array of proteins involved in critical specific biological functions, including neuronal development, regulation of growth, immune response, and lipid and metal binding. Data from this study are accessible from the ProteomeXchange repository (identifier PXD051701). Our findings highlight the presence of small proteins that play key roles in metabolic processes and biosynthetic modulation. In vitro outgrowth experiments with neural stem cells (NSCs) showed that 100 kDa protein extracts from the brain resulted in a greater increase in neurite length compared to the liver and organ mixture extracts. The protein networks identified in the NOP ultrafiltrates may significantly improve biological therapeutic strategies related to neural differentiation and outgrowth. This comprehensive proteomic analysis of 100 kDa ultrafiltrates revealed a diverse array of proteins involved in key biological processes, such as neuronal development, metabolic regulation, and immune response. Brain-specific extracts demonstrated the capacity to promote neurite outgrowth in NSCs, suggesting potential application for neuroregenerative therapies. Our findings highlight the potential of small proteins and organ-specific proteins in the development of novel targeted treatments for various diseases, particularly those related to neurodegeneration and aging.

## 1. Introduction

Our earlier proteomic analysis of 50 kDa organ-specific protein ultrafiltrates (OSPU) from rabbit organs was focused specifically on proteins in the 50 kDa range [1]. We seek to unlock the therapeutic potential of these NOPs, i.e., proteins, small proteins (SPs) and peptides, that could play crucial roles as intracellular modifiers to influence cellular physiology and regenerative ability. These molecules could be especially important as potential therapeutic agents for various medical conditions. Our methodology for examining the protein composition of these NOP ultrafiltrates allows unique insights into the molecular dynamics that govern both physiological and pathological states. This approach not only helps to identify potential therapeutic targets and signaling molecules but also highlights the broader spectrum of organ-specific proteins (OSPs) that could play a role in tissue regeneration and disease treatment [2].

The therapeutic utilization of OSPs has evolved over several decades [3]. Clinically, OSPs have been deployed for their antimicrobial, antioxidative, and immunomodulatory properties. For example, peptides derived from the thymus have been instrumental in restoring T-cell functions, while those from placental extracts and protein hydrolysates have potent antioxidant activities [4,5]. These SPs are particularly valuable for targeting organ-specific conditions, making them suitable for treating diseases or managing conditions related to aging. Proteins in the 100 kDa size range are being investigated for potential therapeutic use in oncology and enzyme replacement therapies, with some therapeutics already in use [6]. This protein size is ideal for penetration and retention in brain tissue [7]. Several therapeutic proteins in the 100 kDa range, such as fusion proteins and antibody fragments, have already been approved by the FDA for various clinical indications. Hence, the 100 kDa cutoff employed during the preparation of ultrafiltrates was chosen due to the proven efficacy of proteins of this size for penetrating into the brain.

Recent advances in bioinformatics and synthetic biology are set to propel the development of novel protein therapies, thus expanding the scope and efficacy of these biologics. Our research has highlighted the potential of 100 kDa protein ultrafiltrates to serve as a foundation for therapeutic strategies that harness the unique properties of proteins, peptides, and potentially protein fragments. By profiling the proteins found within ultrafiltrates, we aim to identify new therapeutic targets and signaling molecules that could revolutionize treatment paradigms.

A major methodological change from conventional approaches that concentrate on isolated neurotrophins or fractionated brain components is represented by the study of unfractionated brain lysates or extracts. Conventional methods in neurotrophin research have frequently depended on investigations of isolated proteins or enrichment techniques, which may restrict a thorough examination of the intricacy of brain tissue.

More recent research shows that enrichment-based methods are still widely used. For instance, rather than concentrating on global proteome interactions, proteomic studies of neurotrophic substances such as trans-Banglene (t-BG) have mainly compared particular effects to isolated NGF protein therapies [8]. Although useful for certain mechanistic enquiries, these focused methods might overlook larger neurotrophin-dependent protein networks. Current proteomic approaches typically employ various fractionation techniques to simplify the complex brain proteome; however, the fractionation has its downsides. For example, one popular method for spatial proteomics is serial ultracentrifugation, which separates frozen postmortem brain tissue into subcellular compartments such as the cytosol, mitochondria, synapse, and nucleus [9]. Although this technique makes it possible to assign hundreds of proteins to physiological compartments, it may also interfere with the natural protein interactions that occur across these boundaries.

Protein–protein interactions that might be broken during fractionation processes are preserved in unfractionated lysates. Even emerging proteome techniques, such as BONCAT analysis, can successfully isolate and identify over 7400 proteins from unfractionated cell lysates [10], proving that complex protein mixtures can be successfully analyzed without extensive fractionation, as shown by in-depth quantitative proteomic studies. Whole-tissue global proteome analysis can uncover surprising relationships between seemingly unconnected pathways. Proteomic screening of the effects of t-BG in PC-12 cells, for example, surprisingly showed alterations in iron-binding proteins that would probably go unnoticed in targeted approaches that only focus on traditional neurotrophic pathways.

Researchers may identify more extensive protein interaction networks by examining unfractionated brain lysates as opposed to isolated neurotrophins or subcellular fractions. This could lead to the discovery of new links between neurotrophic signaling and more general cellular functions.

The therapeutic landscape is likely to undergo significant transformation, especially with the integration of biological therapeutics such as OSPs into clinical practice. These proteins are increasingly recognized for their role in treating a diverse array of conditions, including cancer, diabetes, autoimmune disorders, neurodegenerative diseases, and cardiovascular ailments [11,12]. The potential application of OSPs in novel therapeutic interventions underscores a growing trend towards more targeted and personalized medical treatments.

Our research highlights the immediate implications of identifying and characterizing OSPs and also provides a broader picture of their future role in biomedicine. As the intricate details of these protein networks continue to be revealed, the insights gained could lead to groundbreaking advances in biological therapeutics. The power of peptides and small proteins can thus be leveraged to address some of the most pressing health challenges of our time.

We hypothesize that organ-specific 100 kDa ultrafiltrates contain unique proteins and protein networks that can modulate the differentiation and outgrowth of neural stem cells (NSCs). Brain-derived ultrafiltrates are expected to exhibit the most pronounced effects due to the presence of neuro-specific proteins. This hypothesis is grounded in prior evidence of the functional specificity of SPs and peptides in biological systems, including cell differentiation and growth, as well as antioxidative and immunomodulatory properties. We aim to identify distinct molecular networks and pathways associated with neural differentiation and regenerative processes by examining the proteomic composition and bioactivity of ultrafiltrates derived from different organs.

## 2. Results

### 2.1. Characterization of 100 kDa Ultrafiltrates from Post-Natal Rabbit Brain, Liver and OM

**Figure 1:**
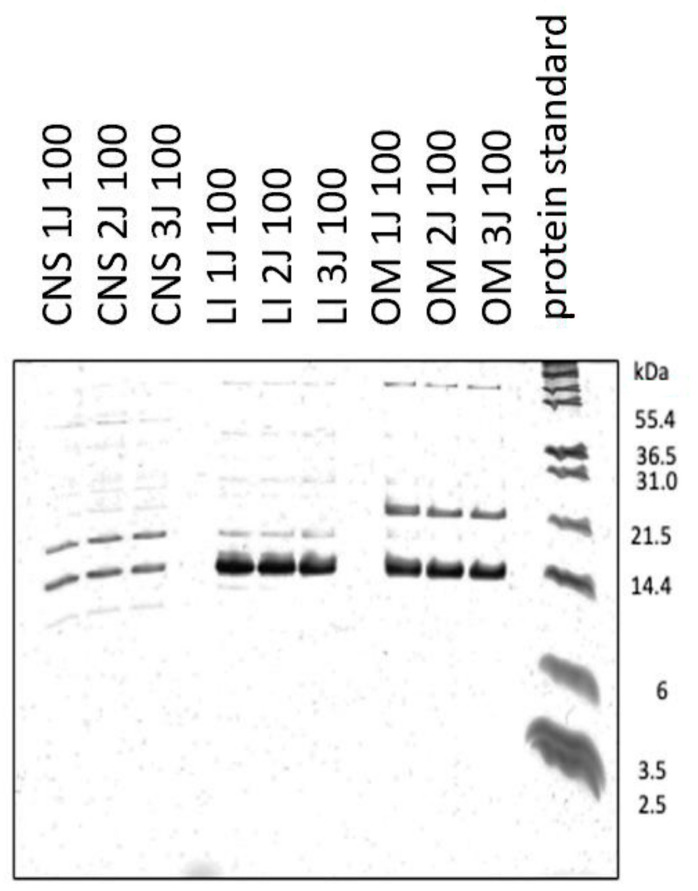
Tris-Tricine-PAGE analysis of three independent samples of 100 kDa ultrafiltrates from the brain (CNS), liver (LI) and organ mixture (OM). The right side shows the M12 protein standard.

Our analysis revealed a notable abundance of protein bands in the 14 kDa range for brain/central nervous system (CNS) and liver, and up to 21.5 kDa for OM (Figure 1).

**Figure 2:**
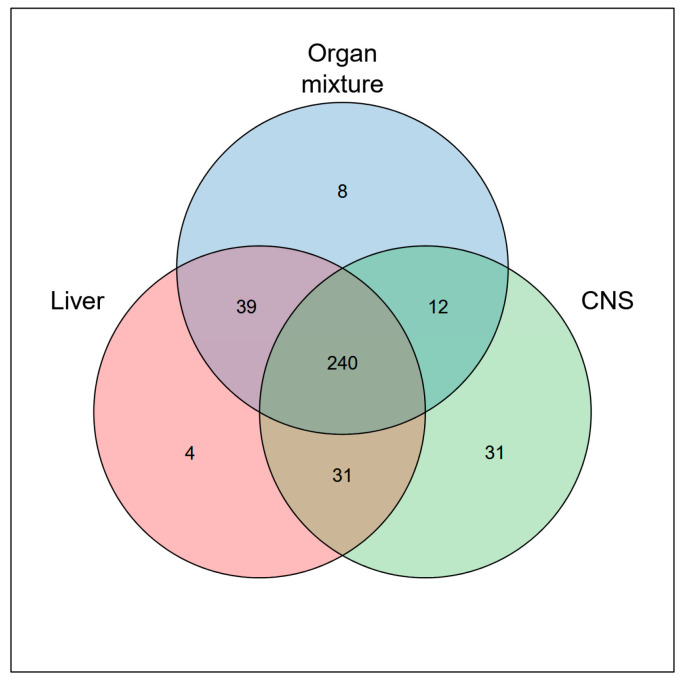
Venn diagram showing overlap of OM, liver and CNS proteins. Numbers represent the number of proteins that are present in two different samples, or in all samples.

For comparative purposes across the three experiments, a Venn diagram of ultrafiltrates from the brain, liver, and OM was generated to visualize the overlap of proteins identified in at least one sample from each experiment (Figure 2). The analysis revealed that a total of 365 proteins were detected in at least one experiment, demonstrating substantial overlap among the datasets. Notably, 240 proteins (65.75%) were found in at least one tissue across all datasets. Additionally, a significant number of unique protein categories and proteins were identified in each organ-specific proteome ultrafiltrate, particularly in the CNS, indicating the presence of distinct organ-specific protein profiles. Supplementary Table S1 lists all the tissue-specific genes related to each sample.

**Figure 3:**
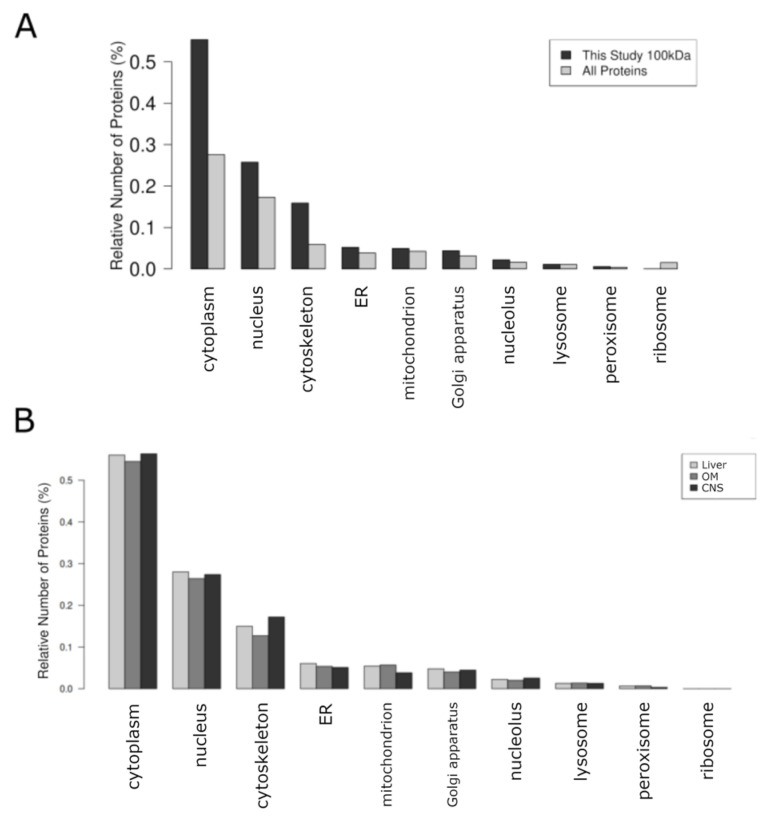
Comparison of organelle-based distribution of proteins measured in this experiment with the general distribution of all proteins. (**A**) “All proteins” represents all known proteins for this species as provided by UniProt. “This Study” represents proteins measured in this experiment. To be able to compare the organelle-based distribution of the proteins from this study with all proteins relative values have been used, otherwise the comparison of the results would be inadequate. (**B**) The same distribution is now shown for each OSP100 (Liver, OM, CNS). LI: Liver, CNS: Brain, OM: Organ mix.

The analysis revealed that the majority of proteins were associated with cellular compartments such as the cytoplasm, nucleus, and cytoskeleton, with a smaller proportion linked to mitochondria (Figure 3). To build on these findings, we conducted a detailed analysis of the enriched proteome, including proteins and peptides identified in OSPs from the liver and CNS, as well as an OM comprising liver, pancreas, placenta, stomach, intestinal mucosa, kidney, and eye. This comprehensive analysis was carried out to gain insights into the Gene Ontology (GO) molecular processes involved in OSPs.

### 2.2. The OSPs Showed GO Enrichment for Glutathione Transferase Activity, Fatty Acid Binding, Identical Protein Binding, RNA Binding, and KEGG Pathways

**Figure 4:**
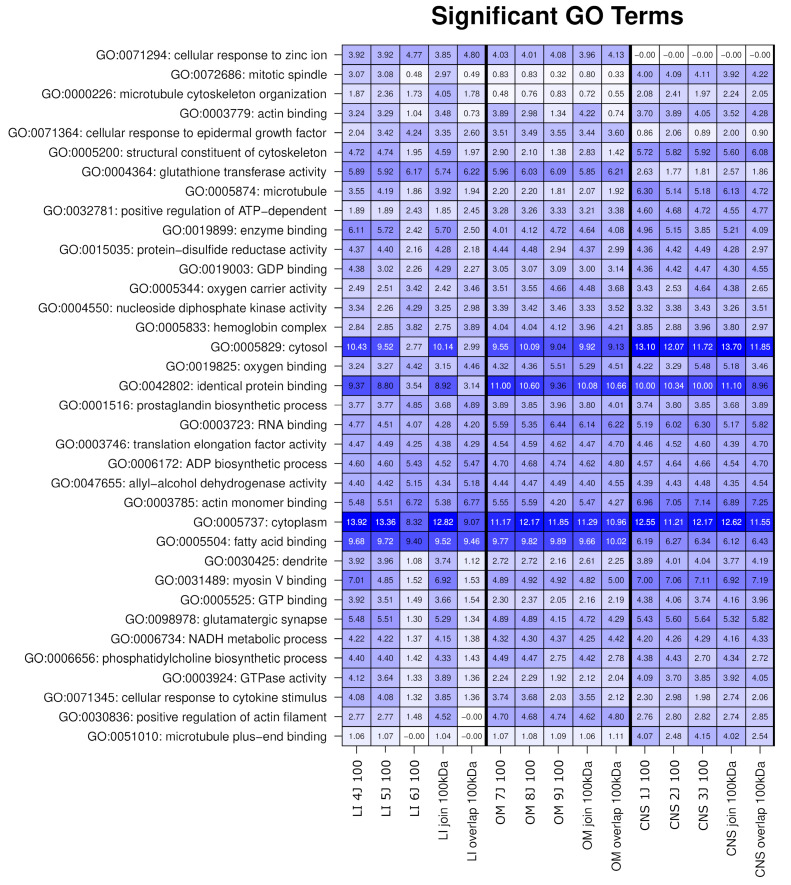
Significant GO terms. The heatmap depicts the significance of Gene Ontology (GO) terms across the various tissues. Each column represents a distinct organ or tissue, while rows correspond to specific GO terms. The intensity of each cell reflects the −log10 (*p*-value) of the enrichment for the GO term in that tissue. The darker the colors, the higher the enrichement. Only the highly significant terms (*p* < 1 × 10^−5^) are displayed. LI: Liver, CNS: Brain, OM: Organ mix.

GO analysis of the OSP100 revealed a highly abundant general GO term related to the cellular component “cytoplasm”, and to the molecular function of “identical protein binding”. A distinct enrichment of proteins predominantly involved in “fatty acid binding”, “cellular response to zinc ion” and “glutathione transferase activity” was observed in the liver and OM (Figure 4).

Highly specific GO terms associated with CNS were identified, including “glutamatergic synapse,” “microtubule plus-end binding,” “actin monomer binding”, and “myosin V”. Supplementary Table S2 presents a comprehensive list of GO terms unique to each tissue.

**Figure 5:**
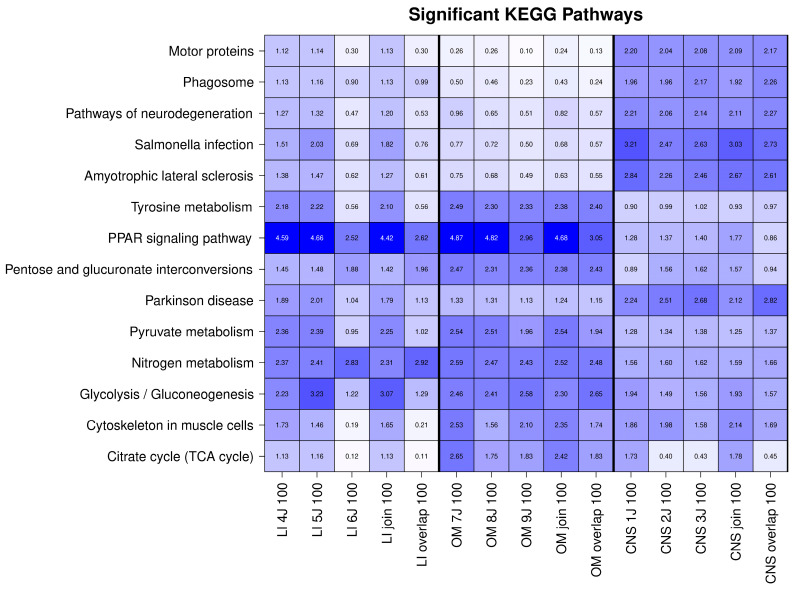
Significant KEGG pathways. Heatmap of significantly enriched KEGG pathways (pFDR < 0.001) in different tissues. Columns represent organs or tissues, and rows correspond to KEGG pathways, with cell colors indicating −log10 transformed *p*-values. The darker the colors, the higher the enrichement. The analysis highlights pathways with significant enrichment in at least one tissue, revealing tissue-specific functional patterns. LI: Liver, CNS: Brain, OM: Organ mix.

KEGG pathway analysis revealed strong enrichment of the PPAR signaling pathway and nitrogen metabolism in OSPs from the liver and OM (Figure 5). Furthermore, both liver and OM OSP100 proteins showed significant enrichment in the glycolysis/gluconeogenesis and tyrosine metabolism pathways. Other notable KEGG pathways primarily associated with CNS OSP100 proteins were linked to neurodegenerative diseases, including terms such as Amyotrophic Lateral Sclerosis (ALS) and Parkinson’s disease (PD). Three most notable KEGG pathways were further analyzed for specific related proteins (see Table 1).

### 2.3. Integrative Analysis Reveals Distinct Functional Profiles Across Organ-Specific Proteomes

**Figure 6:**
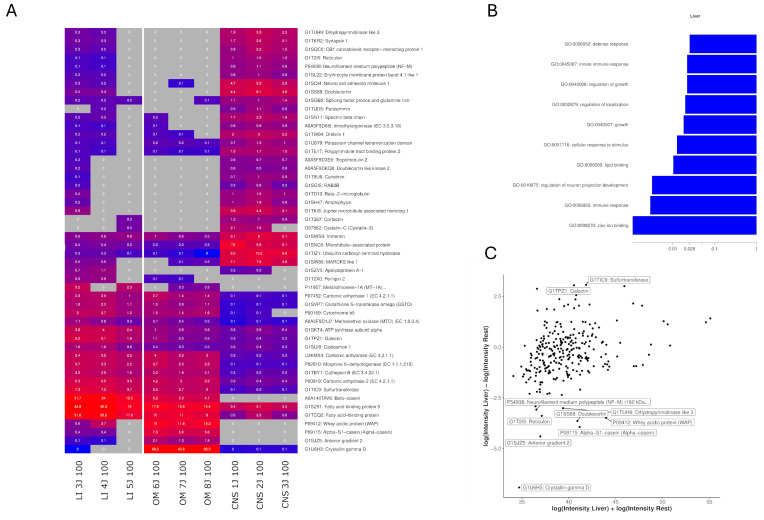
Liver-specific protein analysis. (**A**) Heatmap showing the top 50 proteins with the highest differential expression (highest absolute log2 fold-change) between liver and other tissues. Proteins were included if detected in ≥33% of the liver samples and ≥33% of the other samples. The color intensity represents log-transformed LFQ intensities, with raw values (in millions) annotated. Gray indicates missing data. (**B**) Bar plot of the 10 most significant GO terms enriched in liver tissue, based on proteins with a fold-change > 2. Only proteins detected in this study were used as reference. The *x*-axis shows the enrichment *p*-value obtained by functional enrichment (logarithmic scale). (**C**) MA plot of liver tissue protein quantifications. The *y*-axis shows the log2 fold-change between liver and the other tissues, while the *x*-axis shows the sum of the log2 intensities, corresponding to the total protein abundance. The 10 proteins with the highest differential expression are labeled. LI: Liver, CNS: Brain, OM: Organ mix.

Liver-specific OSPs showed enrichment in GO terms related to “zinc ion binding,” “immune response,” and “regulation of neuron projection development” in the biological process category, and to “glutathione transferase activity” and “fatty acid binding” in the molecular function category (Figure 6B and Supplementary Table S2). To examine the abundance of OSPs in each sample, LFQ intensities were logarithmically transformed, and an MA plot and heatmap were generated for quantification (Figure 6A). A total of 313 proteins identified in the liver samples were analyzed. Of these, 85 were under 20 kDa, 152 were under 50 kDa, and 60 were under 100 kDa, with 17 proteins exceeding the 100 kDa cutoff. Proteins unique to liver OSPs included Ribosome Binding Protein 1, Hydroxyacyl-CoA Dehydrogenase, Alpha-Lactalbumin (Lactose Synthase B Protein) and HCV F-Transactivated Protein 1. Proteins with the highest expression in the liver compared to other tissues included Sulfurtransferase (log2 FC = 3.07 [1.61, 4.57]; Wilcoxon *p*-value: 0.024), Galectin (log2 FC = 3.05 [2.23, 3.83]; Wilcoxon *p*-value: 0.024), fatty acid-binding protein (log2 FC = 3.00 [1.59, 4.43]; Wilcoxon *p*-value: 0.024), Perilipin 2 (log2 FC = 2.88 [2.76, 2.99]; Wilcoxon *p*-value: 0.33), and Metallothionein-1A (log2 FC = 2.77 [2.23, 3.30]; Wilcoxon *p*-value: 0.33) (Figure 6C). The most downregulated proteins in the liver were those found in CNS samples, including Doublecortin (log2 FC = −3.64 [−3.44, −3.86]; Wilcoxon *p*-value: 0.2), Crystallin gamma D (log2 FC = −6.93; Wilcoxon *p*-value: 0.29) and Synapsin I (log2 FC = −2.80 [−2.48, −3.09]; Wilcoxon *p*-value: 0.2).

**Figure 7:**
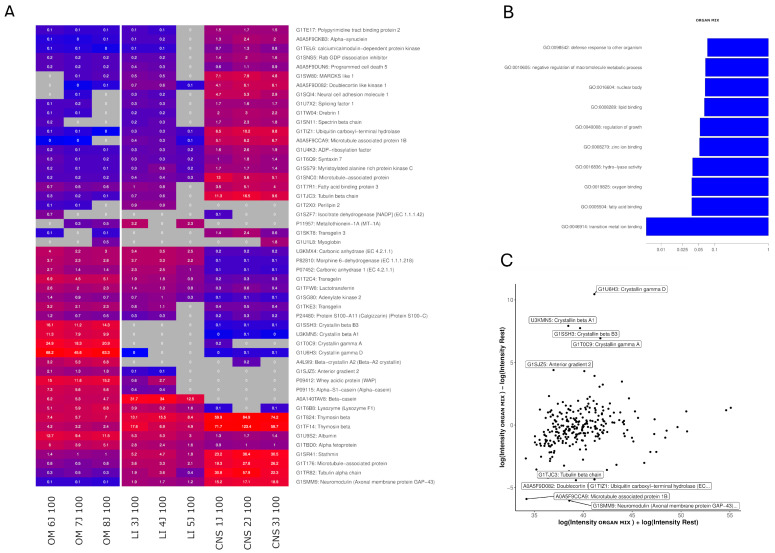
OM-specific protein analysis. (**A**) Heatmap showing the top 50 proteins with the highest differential expression (highest absolute log2 fold-change) between OM and other tissue samples. Proteins were included if detected in ≥33% of liver samples and in ≥33% of other samples. The color intensity represents log-transformed LFQ intensities, with raw values (in millions) annotated. Gray indicates missing data. (**B**) Bar plot of the 10 most significant GO terms enriched in OM samples, based on proteins with a fold-change > 2. Only proteins detected in this study were used as reference. The *x*-axis shows the enrichment *p*-value obtained by functional enrichment (on a logarithmic scale). (**C**) MA plot of OM protein quantifications. The *y*-axis shows the log2 fold-change between OM and the other tissues, while the *x*-axis shows the sum of the log2 intensities, corresponding to the total protein abundance. The 10 proteins with the highest differential expression are labeled. LI: Liver, CNS: Brain, OM: Organ mix.

For OM samples, the most significant GO terms in the biological product and molecular function were “transition metal ion binding”, “fatty acid binding”, “hydro-lyase activity” and “cellular response to zinc ion” (Figure 7B, Supplementary Table S2). A total of 299 proteins were identified, of which 154 unique proteins were related to tissue types present in the OM OSP, i.e., 34 were related to the colon, 8 to the eye, 69 to the kidney, 42 to the liver, 40 to the placenta, and 31 to the small intestine. Of the 299 identified proteins, 77 were under 20 kDa, 154 were under 50 kDa, and 52 were under 100 kDa. High expression of Crystallin proteins (a significant part of the eye lens) was observed, with the highest being Crystallin gamma D (log2 FC = 10.45 [9.79, 11.22]; Wilcoxon *p*-value: 0.06), Anterior gradient 2 (log2 FC = 4.40 [3.82, 4.98]; Wilcoxon *p*-value: 0.2), Whey acidic protein (log2 FC = 3.48 [2.26, 4.7]; Wilcoxon *p*-value: 0.2) and Transgelin (log2 FC = 3.07 [2.19, 3.94]; Wilcoxon *p*-value: 0.02) based on the MA plot (Figure 7C) and heatmap shown in Figure 6A. The lowest expression in comparison to other analyzed tissues was observed for brain-associated proteins including Neuromodulin (log2 FC = −6.03 [−4.87, −7.68]; Wilcoxon *p*-value: 0.02), microtubule-associated protein 1B (log2 FC = −5.91 [−3.91, −7.68]; Wilcoxon *p*-value: 0.02) and Transgelin 3 (log2 FC = −3.99 [−3.29, −4.69]; Wilcoxon *p*-value: 0.2).

Another notable protein detected in the OM was Lysozyme F1 (log2 FC = 3.23 [1.48, 5.29]; Wilcoxon *p*-value: 0.036). While also present in the liver, this protein showed significantly higher expression levels in the OM OSP. The proteins found only in OM samples are shown in Table 2.

The CNS OSPs exhibited significant enrichment in Gene Ontology (GO) terms related to “postsynaptic density,” “response to peptide,” “cellular response to peptide,” and “regulation of neuron projection development” in the biological process category, and to “glutathione transferase activity” and “fatty acid binding” in the molecular function category (Supplementary Table S2). A total of 314 proteins identified in the CNS samples were analyzed, of which 77 proteins were under 20 kDa, 150 were under 50 kDa, 65 were under 100 kDa, and 22 exceeded the 100 kDa cutoff used during production. Proteins exclusively found in CNS OSPs are listed in Table 2. Proteins that were highly expressed in the CNS samples included microtubule-associated protein (log2 FC = 4.83 [4.16, 5.5]; Wilcoxon *p*-value: 0.02), Neuromodulin (log2 FC = 5.59 [4.11, 7.07]; Wilcoxon *p*-value: 0.02), Doublecortin (log2 FC = 3.64 [3.44, 3.86]; Wilcoxon *p*-value: 0.2), Reticulon (log2 FC = 3.39 [3.12, 3.65]; Wilcoxon *p*-value: 0.2), Synapsin I (log2 FC = 2.80 [2.48, 3.09]; Wilcoxon *p*-value: 0.2) and Neural Cell Adhesion Molecule (log2 FC = 4.79 [4.02, 5.71]; Wilcoxon *p*-value: 0.1). The lowest expression was observed mainly for Crystallin proteins, especially Crystallin beta A1 (log2 FC = −7.92 [−7.54, −8.26]; Wilcoxon *p*-value: 0.1), and Lysozyme (log2 FC = −5.61 [−4.99, −6.19]; Wilcoxon *p*-value: 0.07).

Table 2 highlights key proteins identified in the CNS ultrafiltrates and their specific roles in neuronal development and axonal extension. These molecules constitute a complex functional network that collectively enhances neurite formation and elongation.

Table 3 identifies proteins involved in selected KEGG pathways related to neuronal outgrowth.

**Figure 8:**
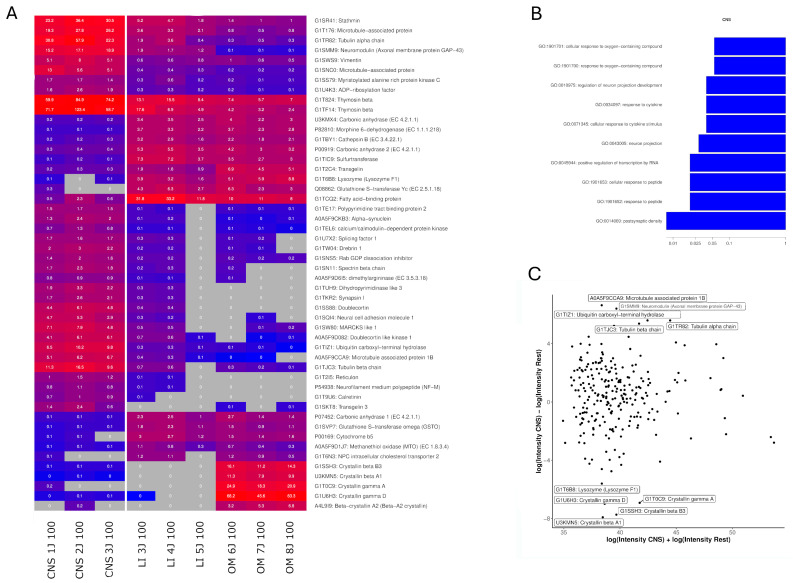
Brain-specific protein analysis. (**A**) Heatmap showing the top 50 proteins with the highest differential expression (highest absolute log2 fold-change) between brain and other tissue samples. Proteins were included if detected in ≥33% of liver samples and ≥33% of the other samples. The color intensity represents log-transformed LFQ intensities, with raw values (in millions) annotated. Gray indicates missing data. (**B**) Bar plot of the 10 most significant GO terms enriched in brain tissue samples, based on proteins with a fold-change > 2. Only proteins detected in this study were used as reference. The *x*-axis shows the enrichment *p*-value obtained by functional enrichment (on a logarithmic scale). (**C**) MA plot of brain tissue protein quantification. The *y*-axis shows the log2 fold-change between brain and the other tissues, while the *x*-axis shows the sum of the log2 intensities, corresponding to the total protein abundance. The 10 proteins with the highest differential expression are labeled. LI: Liver, CNS: Brain, OM: Organ mix.

The GO classifications for each OSPs revealed distinct molecular signatures (Figure 6, Figure 7 and Figure 8, Table S2), while also showing some overlap of GO terms. This overlap is due to shared proteins or protein groups, suggesting joint involvement in overlapped GO terms such as “axon extension” in liver and brain, or “cellular response to zinc ion” in liver and OM. There was no significant overlap between CNS and OM. The proteomics analysis revealed an enrichment of key functional proteins across rabbit organs, as supported by GO term analysis.

### 2.4. Identification of Highly Expressed Small Proteins

Our analysis revealed a subset of proteins that was present across all examined tissues, albeit at varying levels of expression. These ubiquitous proteins, totaling 65 unique entities, constituted 17.81% of the overall protein catalog of 365 proteins. They were most pronounced in hepatic samples (19.75%), and least prominent in cerebral tissue (17.52%).

**Figure 9:**
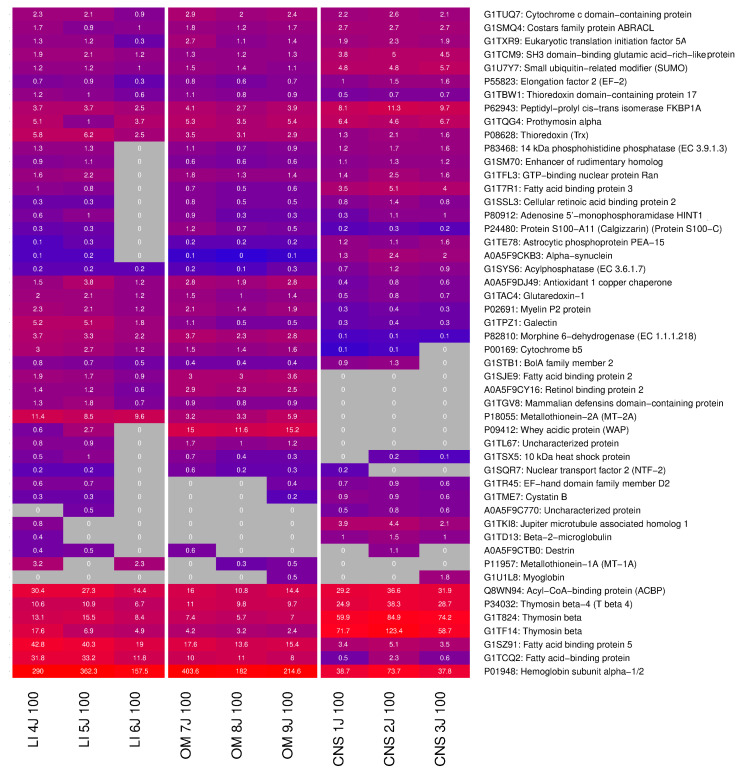
Heatmap of the top 50 small proteins expressed at high levels in all samples (<150 amino acids). Gray color indicates missing values (protein not measured). Protein quantification values are annotated in the cells (LFQ/1M). LI: Liver, CNS: Brain, OM: Organ mix.

Figure 9 shows the top 50 of these widely distributed proteins, specifically those with <150 amino acids, and ranked by their expression level. A comprehensive inventory is provided in the Supplementary Materials (Supplementary Table S3).

Of note, 37% of these proteins were consistently detected across all tissue types. In contrast, only three proteins exhibited tissue exclusivity: two in liver samples and one in CNS. This observation suggests that proteins unique to specific tissues tend to be larger, exceeding 150 amino acids in length.

Despite their widespread occurrence, certain proteins displayed a marked tissue preference in terms of their abundance. For example, cerebral tissue showed elevated levels of two specific proteins involved in cellular regulation. Hepatic samples were characterized by high concentrations of a lipid-binding protein, while a protein associated with immune function was particularly abundant in samples derived from the oral mucosa.

As shown in Table 4, CNS samples contained the largest number (*n* = 29) of uniquely occurring proteins. Liver samples, on the other hand, had only four detectable unique proteins.

### 2.5. CNS Extracts of 100 kDa Stimulate Neurite Outgrowth of NSCs

**Figure 10:**
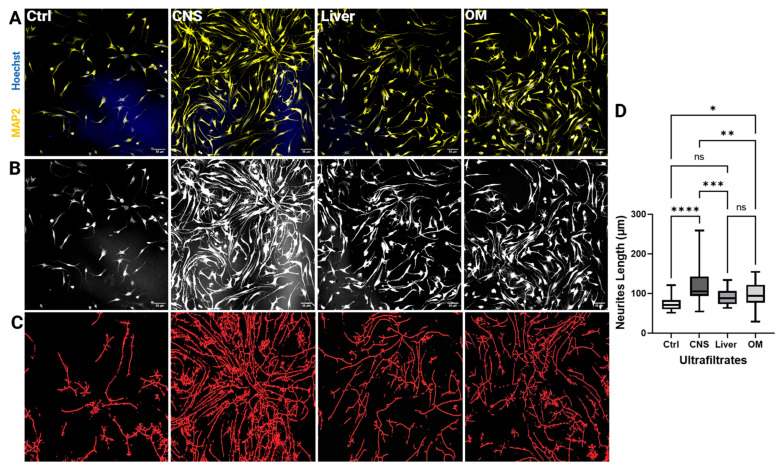
Measurement of neurite length. (**A**) NSCs treated with ultrafiltrates (Ctrl, CNS, Liver, and OM) were immunostained for MAP2 (yellow), while the nuclei were stained with Hoechst 33,342 (blue). Scale bar: 50 μm. (**B**) Black and white micrographs showing the growth of neurites after treatment of NSCs with ultrafiltrates: Ctrl, CNS (brain), Liver, and OM (organ mixture). Scale bar: 50 µm. (**C**) Skeletonized micrographs for a zoomed-in view of NSCs in the figures shown in (**B**), the red color represents the branches of Neurons. (**D**) The boxplot shows the neurite length in μm of 30 different cells from each of the four groups (Ctrl, CNS, Liver, and OM). Measurements shown are the mean ± SD; *, *p* < 0.05; **, *p* < 0.01; ***, *p* < 0.001; ****, *p* < 0.0001; ns, not significant. OM: Organ Mix.

Figure 10 shows the NSC’s growth after ultrafiltrate treatments in the four different groups. The treatment of NSCs with brain extracts showed higher cell density on day 8 than other groups (Figure 10A,B). Skeletonized micrographs for each treatment group showed fiber density and branches of neurites in Figure 10C. Neuronal cells were mostly bipolar and NSCs treated with brain UFs showed the highest fiber density. The overlapping and intermingling fibers could not be quantified related to the numbers per cell because it is difficult to determine which dendrite belongs to which soma. In this experiment it was found that brain extracts significantly promote neurite elongation, with the average neurite length being substantially higher compared to NSCs treated with liver extracts or a mixture of organ extracts. Specifically, treatment with brain extract resulted in a mean neurite length of 121.4 ± 42.77 μm, compared to 89.80 ± 18.41 μm for liver extract and 96.8 ± 31.98 μm for the organ mixture extract (Figure 10D). Statistical analysis confirmed that the differences in neurite length between the brain extract treatment and the other treatments were highly significant (*p* < 0.05), validating the robustness of our research methodology with the neural outgrowth assay. Additional observations indicated that NSC viability and proliferation rates were comparable across all treatment groups, including brain, liver, and organ mixture extracts. This suggests the observed differences in neurite outgrowth were not due to variations in cell viability or proliferation but were specifically attributable to distinct properties of the brain extract that enhance neurite extension. Together, these findings highlight the unique potential of brain extracts in promoting neurite outgrowth in NSCs. However, we believe that further analysis should be conducted to accurately determine the number of dendrites per cell in addition to electrophysiological properties for each treatment group.

## 3. Discussion

### 3.1. Efficacy of CNS Extracts

In accordance with their enhanced neurodevelopmental proteins, brain-derived ultrafiltrates showed greater neurite outgrowth stimulation (121.4 μm vs. 89.8–96.8 μm for liver/OM). The increase in neuronal outgrowth by the ultrafiltrates from the brain could be based on various mechanisms of the peptides and proteins found in the ultrafiltrates as shown in Table 2.

Growth cone motility is directly supported by neuromodulin (GAP-43) [31] and doublecortin [20], which control actin/microtubule dynamics. Synapsin I and Neural Cell Adhesion Molecule (N-CAM) facilitate axon guidance and synaptic connection. Among the proteins identified in the 100 kDa brain extracts, Neural Cell Adhesion Molecule (N-CAM) stands out as a pivotal factor in neuronal growth and connectivity [32]. The role of N-CAM in mediating cell–cell adhesion is essential for the structural integrity and stability of developing neurons, allowing them to interact with other neurons and supporting cells. The adhesive properties of N-CAM not only provide a scaffold for neuronal growth but also facilitate axon guidance, thus ensuring that growing axons navigate properly through the extracellular environment toward their target destinations. This observation might be of interest for in vivo experiments related to brain damage and repair. Additionally, Kolkova et al. [13] found that NCAM causes prolonged MAP kinase activity, which is necessary for neural development, via activating the Ras-MAP kinase and FGF receptor-PLCγ-PKC pathways. This is consistent with our KEGG pathway analysis, which identified cytoskeletal regulation and neurodegenerative disease pathways (such as Parkinson’s and ALS) as being essential for neurite elongation (Table 1 and Table 2).

The activity of proteins such as GAP-43, Thymosin Beta-4, and calretinin ensures that the growth cone can effectively navigate its environment. These findings are consistent with previous research [33], which showed that GAP-43 phosphorylation through fibroblast growth factor (FGF) receptor activation is necessary for NCAM-stimulated neurite outgrowth. Their findings are also supported by our discovery of the FGF receptor’s role in GAP-43 phosphorylation, pointing to a signaling pathway that is constant across species and experimental paradigms.

The superior neurite outgrowth induced by brain extracts (121.4 μm vs. 89.8–96.8 μm for liver/OM) underscores their therapeutic potential, akin to natural compounds like Ginkgo biloba extract (GBE). Similar to our brain extract results, Lejri et al. demonstrated that GBE increases neurite length in SH-SY5Y cells by 77% via activating the Akt/mTOR pathway [34]. This implies that phytochemicals and organ-specific protein networks may enhance neuroregenerative effects. Furthermore, by sequestering Aβ oligomers, EV-associated prion protein (PrPC) was identified by Quiroz-Baez et al. in their review as a neuroprotective agent in Alzheimer’s models. The discovery of comparable EV-enriched proteins in brain ultrafiltrates, such as ATP synthase β-subunit, suggests that there are common strategies for preventing protein aggregation in neurodegeneration [35].

Collectively, these proteins contribute to the finely tuned regulation of growth cone dynamics and cytoskeletal remodeling, which are fundamental to axon extension and neuronal outgrowth [36]. The growth cone is located at the tip of developing axons and is highly responsive to extracellular guidance cues. It is possible that these observed effects are mostly concentration dependent. This should be further investigated, along with the ideal concentration for the whole CNS OSP100.

### 3.2. The Efficacy of Liver and OM Extracts

Although less neuroactive, liver extracts demonstrated metabolic dominance (e.g., Sulfurtransferase, fatty acid-binding protein), indicating functions in lipid metabolism and detoxification. The intermediate potency of OM was probably caused by a combination of metabolic/structural elements from non-neural tissues (eye crystallins, Lysozyme F1) and neurosupportive proteins (Transgelin, Anterior Gradient 2). Even in the absence of brain-level specialization, the OM’s partial effectiveness points to combinatorial effects from cross-tissue proteins.

The neurite-promoting ability of the OM (96.8 μm) might result from common cytoskeletal regulators, synergistic metabolic factors and an abundance of structural proteins.

All tissues have ubiquitous proteins such as microtubule-associated proteins (RP/EB family) and Profilin, which serve as a foundation for neurite elongation. Improved energy metabolism or redox balance in NSCs may be achieved by enriched OM pathways (such as glycolysis and transition metal binding). Crystallins, which are prevalent in OM, are known to maintain neurite integrity indirectly by stabilizing cell membranes under stress [26].

ARHGDIB and RHOA, found exclusively in OM samples, were not present in CNS samples. Through the maintenance of these signaling molecules in their inactive GDP-bound state, ARHGDIB, a Rho GDP-dissociation inhibitor, regulates Rho GTPase activity [37]. RHOA’s known involvement in neurite suppression through actin cytoskeleton rigidification and microtubule stabilization interacts with this regulatory function [38,39]. The OM’s efficacy may be due to ARHGDIB’s ability to reduce RHOA activity through GTPase sequestration, even though brain extracts showed higher neurite-promoting capacity. The exact stoichiometric balance of brain-derived regulators, specifically the co-occurrence of ARHGDIB with its binding partners like p75 neurotrophin receptor [40] and downstream effectors like PAK3 (Table 2), is probably absent from the protein composition of the OM. The significance of tissue-specific molecular networks in neuroregulatory capacity is highlighted by the possibility that this imbalance may lead to less-than-ideal coordination between cytoskeletal remodeling processes and RhoA inactivation.

### 3.3. Wider Outcomes for Neuroregenerative Therapy

Several proteins are known to have an impact on neuronal outgrowth [30]. These include Profilin, which acts as a key regulator of actin dynamics to ensure that a balance is maintained between the pool of readily available actin monomers and the promotion of controlled filament assembly [31]. This precise regulation is essential for the complex and dynamic processes involved in neuronal outgrowth, including growth cone guidance, neurite formation, and synapse development. The microtubule-associated protein RP/EB family member 2 protein is involved in microtubule dynamics and stabilization, which are crucial for neurite outgrowth and neuronal migration [32]. This protein helps to regulate microtubule growth at the plus ends, thus ensuring the proper formation of neurites. The protein alpha-tubulin N-acetyltransferase 1 (alpha-TAT1) acetylates alpha-tubulin, which is a post-translational modification that enhances microtubule stability [33]. Stable microtubules are essential for the structural integrity of growing neurites, thus promoting neurite elongation. The protein ENAH Actin Regulator, also known as Mena, is known to regulate actin dynamics [34]. This protein is fundamental for growth cone movement and neurite outgrowth, and it plays a critical role in actin polymerization and cell motility. Profilin binds to actin monomers (G-actin) and facilitates their addition to the growing actin filament (F-actin), thereby regulating actin polymerization [12]. Actin dynamics are essential for the extension and branching of neurites. Similarly to RP/EB family member 2, the microtubule-associated protein RP/EB family member 3 protein is also involved in the regulation of microtubule dynamics [35]. It assists in the stabilization and proper functioning of microtubules, thereby contributing to neuronal growth and guidance. The abovementioned proteins are integral to regulation of the cytoskeleton, which is crucial for neurite outgrowth and neuronal development. The roles played by these proteins in microtubule and actin filament dynamics highlight their importance in the structural and functional maturation of the neuron cytoskeleton. This in turn is crucial for neurite outgrowth and neuronal development.

In disorders like spinal cord injury or neurodegenerative illnesses, brain-specific proteins (such as Doublecortin and Neuromodulin) are effective options for promoting axonal regeneration. Neurodegenerative diseases such as PD, Alzheimer’s disease (AD) and Huntington’s disease (HD) share common pathological features such as neuronal degeneration and protein aggregation [41]. Several key proteins identified through KEGG pathway analysis play crucial roles in these conditions (see Table 2). For example, although primarily associated with ALS, FUS protein also impacts AD and PD through its aberrant aggregation and disruption of RNA metabolism. Similarly, mutations in VAPB play a role in ALS and affect intracellular transport and ER stress but also contribute to neurodegeneration in PD and frontotemporal dementia. The neurofilament proteins NF-M and NF-H have been implicated in various neurodegenerative disorders, where their abnormal aggregation correlates with other disease-specific pathologies such as tau in AD and alpha-synuclein in PD [42]. Profilins (PFN1 and PFN2) regulate actin dynamics, and their dysfunction affects synaptic plasticity and neuronal morphology in multiple diseases [42]. Tubulins are essential for microtubule stability and are disrupted in AD and PD, thereby impacting intracellular transport and contributing to neuronal loss [43]. The discovery of brain-specific proteins like Synapsin I and Doublecortin (DCX) is consistent with results from PPI network investigations in neuroregeneration. Issa et al. [44], for example, highlighted proteins including IL6 and CXCL12 and identified NGF, BDNF, and GDNF as key nodes in neural repair networks. By identifying tissue-specific enrichment of Reticulon (endoplasmic reticulum shape) and Calretinin (calcium signaling), our study broadens this field. These two proteins may work in concert with classical neurotrophic factors to improve synaptic integrity and axonal guidance. Interestingly, crystallins [45] and transgelin are responsible for the intermediate effectiveness of organ mixture (OM) extracts, which is similar to the combinatorial effects seen in systems biology models where regenerative outcomes are driven by multi-protein interactions.

### 3.4. Mechanistic vs. Network-Oriented Analysis

The basic ideas of systems biology, which acknowledges that biological functions originate from the dynamic interactions of molecular networks rather than from isolated protein activities, are best illustrated by our study [46,47]. The emergent properties of protein networks—aspects that result from the combined behavior of interconnected components but cannot be predicted by examining individual proteins separately—are acknowledged by this systems-level approach [48,49]. These emergent characteristics in biological systems include self-sustaining feedback loops, the creation of different outputs based on the strength and duration of inputs, and the integration of signals across multiple time scales.

One of the best examples of a complex biological system where complex networks of protein interactions give rise to emergent behaviors is the brain [50,51]. About 70% of brain proteins function in stable complexes, according to recent developments in network medicine [51,52]. This highlights the fact that biological systems are made up of interconnected modules rather than separate parts. These native protein–protein interactions, which could be broken during traditional fractionation processes, are preserved by our method of examining unfractionated brain ultrafiltrates [53].

This network-centric viewpoint is consistent with the current understanding that system function cannot be understood as the sum of its parts because cellular functions are the result of non-linear interactions between components [54]. An example of how emergent properties of intact protein networks can produce effects that surpass those predicted from individual protein studies is the neurite outgrowth enhancement seen in our brain OSP100 (121.4 μm vs. 89.8–96.8 μm for liver/OM).

Because native protein interaction networks drive biological effects through synergistic pathways that isolated protein studies are unable to capture, our findings highlight the vital necessity of analyzing unfractionated lysates. Vaudel’s evidence that more than 70% of brain proteins function in stable complexes supports the idea that biological systems function through interconnected modules rather than isolated components [55], as illustrated by Barabási’s network medicine framework [56]. Reductionist methods ignore emergent characteristics like the synergistic anti-apoptotic effects of neurotrophin combinations and run the danger of incorrect attribution, as demonstrated by BDNF’s reliance on sortilin for optimal trafficking. The advantage of whole-lysate analysis is consistent with Parkinson’s studies indicating α-synuclein oligomer network reliance and Alzheimer’s biomarker studies displaying disease symptoms in network disruptions. Although targeted validation is useful, it is against systems biology principles and NIH standards for studying biological complexity to need specific protein attribution for complicated lysate effects. Our observed neurite outgrowth enhancement by brain ultrafiltrates most likely results from coordinated modulation of various targets (e.g., Trk/p75NTR balance) and endogenous regulatory mechanisms conserved in intact networks. This comprehensive approach respects the intrinsic complexity of the brain while addressing the conceptual and technological constraints of evaluating more than 1000 components separately. The onus should be to disprove systemic effects, not vice versa, as ultrafiltrates represent more the brain’s native state [57].

### 3.5. Endogenous Proteolysis as a Functional Network Component

Based on mounting evidence that endogenous proteolysis performs essential functional roles in brain tissue beyond simple protein degradation, we decided to avoid using protease inhibitors during sample preparation [58,59]. In brain neurogenic niches, where molecules produced by particular protease activity can either promote or inhibit neural stem cell proliferation, differentiation, and migration, regulated intramembrane proteolysis has been found to be a key mechanism regulating neurogenesis [60].

Recent research has shown that bioactive protein fragments produced by endogenous proteolytic processing support intercellular signaling and neuroplasticity [61]. During memory formation and retrieval, activity-dependent protein degradation via the ubiquitin–proteasome system plays a crucial role at brain synapses. NMDA-dependent increases in degradation-specific polyubiquitination target proteins involved in synaptic structure and translational control [58]. In addition to being degradative, this proteolytic activity is a key modulator of synaptic plasticity, which is essential for the development of long-term memory [62].

Additionally, brain tissue’s endogenous proteolysis shows tissue-specific patterns that are very different from those of other organs [63]. Proteolytic fragments that preserve functional domains while producing new N-termini through physiological cleavage events were identified by our analysis [61]. These results lend credence to the idea that the natural proteolytic landscape of brain tissue is not an experimental artifact that needs to be removed, but rather a functional element of the neural protein network.

Since endogenous proteolytic networks preserve the physiologically significant protein states that exist in vivo [60], their preservation in OSP100 may therefore be a factor in the observed enhancement of neurite outgrowth. This method reflects the dynamic character of brain protein networks, where functional protein fragments involved in neural development and regeneration are shaped by controlled proteolysis.

### 3.6. Integrating Reductionist and Systems Perspectives

Although a systems biology approach is the focus of our study, it is important to recognize that reductionist and holistic approaches are essentially complementary and interdependent rather than antagonistic [64]. Reductionist methods are still crucial for comprehending particular protein interactions and functions, and they have yielded amazing insights into molecular mechanisms [65]. When applied to intricate biological processes like neurite outgrowth, which entails coordinated actions across several molecular pathways, these methods, however, have inherent limitations.

Examining emergent biological properties highlights the shortcomings of strictly reductionist methods. In complex biological systems, the behavior of the whole cannot be predicted from the characteristics of its constituent parts, and small changes can result in disproportionately large responses [66]. Focusing solely on individual proteins in the context of neuroregeneration runs the risk of omitting network-level interactions that produce emergent neurite-promoting effects [64].

On the other hand, systems approaches have drawbacks as well, especially when it comes to the validation and mechanistic interpretation of particular molecular events [67]. Large datasets without enough mechanistic insights or holistic models that do not match empirical reality can be produced by pure systems-level analyses. While conventional reductionist methods that concentrate on individual proteins are limited in their ability to capture the complexity and interconnectedness of cellular systems, the combination of systems biology and proteomics enables thorough analysis [68].

Our methodology illustrates the potential for synergy between these approaches [52]. Our thorough proteomic characterization of individual proteins (Table 2) offers the mechanistic basis required to comprehend these network effects, while the systems-level analysis of unfractionated ultrafiltrates revealed protein networks linked to neurite outgrowth. This integrated approach recognizes that reductionist findings must be interpreted in the context of intact biological systems, while observations from holistic studies necessitate mechanistic insights obtained from reductionist work [69].

Future research should keep utilizing both viewpoints, utilizing reductionist techniques to confirm particular molecular mechanisms and protein functions within these networks and systems approaches to find emergent properties and network behaviors. The best way to comprehend intricate biological processes like neuroregeneration and create interventions that are therapeutically relevant is through this complementary approach.

### 3.7. Methodological Advances and Limitations

Tris-Tricine-PAGE, LC-MS/MS, and 100 kDa ultrafiltration were used in this analysis, which conforms to accepted procedures for separating small proteins (SPs) or extracellular vesicles (EVs) from biological fluids. The usefulness of this method for neurological research was validated by Noben et al. [70], who showed that centrifugal ultrafiltration in conjunction with LC-MS/MS was effective in discovering new cerebrospinal fluid CSF biomarkers in multiple sclerosis. Similarly, UF-SEC (ultrafiltration-size exclusion chromatography) was emphasized in the article by Kangas et al. as a reliable technique for EV separation from CSF, obtaining proteome profiles even from 0.5 mL samples [71]. By concentrating on organ-specific 100 kDa ultrafiltrates, which filtered out bigger molecules while selecting for bioactive proteins (<50 kDa) essential for neurite outgrowth, our findings go beyond these approaches. This is in contrast to earlier research that frequently focused on EV-associated proteins rather than tissue-specific molecular networks.

There are several inherent limitations when working with natural biological materials. Despite standardization efforts, each extraction process may yield slightly different samples due to the variability in protein expression within living organisms. Such variations can occur across different tissues from the same organism, or between individuals from the same species. To minimize these differences, low temperatures were maintained throughout the sample preparation process to preserve their integrity. In addition, low flow rates, specialized membranes, and appropriate pressures were used to minimize any changes in protein structure.

As our objective was to identify naturally occurring proteins, no additional inhibitors were added during or after sample preparation. By avoiding protease inhibitors, our method maintained native protein profiles; nevertheless, it also increased variability from endogenous proteolysis, a restriction also observed, for example, in CSF EV investigations. To address tissue heterogeneity, future research could incorporate single-cell proteomics or cross-linking techniques. Furthermore, the CSF proteomes enriched in external exosomal proteins contrast with our datasets’ preponderance of cytoplasmic proteins (Figure 3), indicating that organ-specific ultrafiltrates would be better able to capture intracellular modifiers. Translational relevance will become clearer if comparisons are extended to human-derived samples [70].

We have also analyzed two datasets in Mascot using Trypsin, Semi-Trypsin, and no specific enzyme as search parameters. The results indicate a slight increase in the number of identified proteins, suggesting that some mild degradation is present in the samples. However, the increase in protein identifications is not dramatic, implying that the extent of degradation is limited and does not substantially impact the overall protein profile of the extracts (Supplementary Figure S1). Lower molecular weight proteins (<10 kDa) are also not as prone to degradation as higher molecular weight proteins [72].

The effectiveness of ultrafiltration may also be affected by the type of tissue being processed. Its fat content, amount of connective tissue, and cellular density may all affect the diversity and yield of proteins in the ultrafiltrate. Moreover, the amount of vascularization in ultrafiltrated tissue may change the protein profile due to high amounts of blood proteins such as hemoglobin. Liver, brain and parts of the OM (mainly liver and pancreas) are either highly vascularized or have a high lipid content. Despite these characteristics, tissue-specific patterns with varying amounts of the different proteins were observed.

The combination of Tris-Tricine PAGE with LC-MS/MS is a powerful approach for analyzing small proteins. It offers superior resolution for proteins below 30 kDa, and effective separation in the 1–100 kDa range, as proven numerous times [73]. This method demonstrates high sensitivity and is capable of detecting proteins in the low μg/mL range without immuno-enrichment. Furthermore, it allows multiplexed analysis and absolute quantification. However, it is important to be aware of potential biases, including the preferential detection of smaller, hydrophobic proteins, and the limited capture of post-translational modifications in bottom-up approaches. The resolution of this technique can be further enhanced by adjusting the gel composition and adding urea, although this may affect the detection of larger proteins. While highly effective for the analysis of small proteins, users should be aware of limitations such as sample volume constraints and reduced throughput compared to immunoassay-based platforms. Despite these considerations, the Tris-Tricine PAGE and LC-MS/MS combination is a valuable tool for deep proteome analysis and can identify thousands of proteins and peptides in complex samples.

## 4. Materials and Methods

### 4.1. Production of 100 kDa Ultrafiltrates

Postmortem frozen laboratory-grade rabbit pups (no more than −20 °C) were received, thawed, washed, and dried off. The pups obtained were laboratory-grade M91 broiler rabbits maintained in broiler rabbit production systems by interline hybridization from the NZW (New Zealand White) rabbit strain. The rabbits were euthanized under the supervision of a veterinarian in accordance with Act No. 246/1992 Coll., on the Protection of Animals Against Cruelty, paragraph 18, for the purpose of organ collection. Rabbit brain, liver, and an organ mixture (OM; liver, pancreas, placenta, stomach, intestines, kidney, eye) were added to a saline solution. For OM, the wet weight proportions for each organ were equal (1:1:1:1:1:1:1). This was homogenized (laboratory mixer WARING, McConnellsburg, PA, USA) and centrifuged at 6000 rpm (Sorvall RC-5B refrigerated centrifuge, Du Pont, Wilmington, DE, USA) for 10 min in a refrigerated centrifuge. The homogenate underwent gentle prefiltration using ceramic filters (DWK Life Sciences, Mainz, Germany), after which the samples were rapidly frozen at −70 °C for 24 h. Subsequent microfiltration was carried out using a crossflow filtration system (Sartorius Stedim Biotech, Aubagne, France) equipped with molecular weight cutoff (MWCO) filters (Sartorius Stedim Biotech, Aubagne, France) of 300 kDa and 100 kDa. To minimize any changes in protein structure, a hydrosart membrane was used along with a gentle flow rate of 100 mL/min and a transmembrane pressure of 0,6 bar for both the 300 and 100 kDa MWCO membranes. The resulting permeate was collected in sterile medium flasks for further analyses. All procedures were standardized and performed under cold conditions on ice. The concentration of OSPs was quantified using a standard bicinchoninic acid (BCA) assay kit (Merck Millipore, Burlington, Massachusetts, USA) as recommended by the manufacturer. To ensure the validity and reproducibility of the data, three completely independent biological samples (from three different animals) were processed for each organ type (brain, liver, and organ mixture). Each biological sample underwent the complete extraction and analysis protocol separately (see Table 5).

### 4.2. Analysis by Tris-Tricine-Polyacrylamide Gel Electrophoresis

The procedure for sample preparation and protein separation was modified to enhance clarity and efficiency. Samples containing 5 µg of protein in a maximum volume of 12 µL were combined with a custom-made, 4× sample buffer consisting of 250 mM Tris (pH 6.8), 12% glycerol, 4% SDS, 10% β-mercaptoethanol, and 0.05% bromophenol blue. The mixture was then heat-denatured at 95 °C for 5 min (all reagents from Gibco, Frederick, MD, USA).

After cooling, the samples were loaded onto a discontinuous polyacrylamide gel for electrophoresis. The gel system comprised a 4% stacking gel and an 18% separating gel. The stacking gel was prepared using a 29:1 ratio of acrylamide to bisacrylamide, while the separating gel utilized a 32:1 ratio. Both gels contained appropriate concentrations of Tris buffer, SDS, and polymerization catalysts (TEMED and APS). The separating gel also included 14% glycerol to improve resolution.

Electrophoresis was carried out at 150 volts for 3 h using a Mini-protein II Dual Slab Cell system (Bio-Rad, Hercules, CA, USA). Following separation, the proteins were visualized using a mass spectrometry-compatible silver staining protocol (SilverQuest Silver Staining Kit, Thermo Fisher Scientific, Waltham, MA, USA). To determine the molecular weights of the separated proteins, a commercial protein standard Mark12 (Thermo Fisher Scientific, Waltham, MA, USA) was run alongside the samples.

The stained gel was digitally recorded at 150 dpi using a high-resolution scanner equipped with a transparency adapter. This imaging step allowed for subsequent analysis and documentation of the protein separation pattern.

### 4.3. Analysis Using Liquid Proteolysis

Proteolysis in solution was conducted by dissolving 6 µL of protein in 10 µL of 8 M urea. For protein reduction, tris(2-carboxyethyl)phosphine (TCEP) (Thermo Fisher Scientific, Waltham, MA, USA) was added to a final concentration of 5 mM, and the sample was then incubated for 20 min at room temperature. Following reduction, alkylation was carried out by adding iodoacetamide (IAA) (Thermo Fisher Scientific, Waltham, MA, USA) to a final concentration of 10 mM and incubating the reaction mixture in the dark for 20 min at room temperature. The sample was then diluted 1:10 with 50 mM triethylammonium bicarbonate (TEAB) buffer (Thermo Fisher Scientific, Waltham, MA, USA). Proteolytic digestion was initiated by adding 100 ng of trypsin (Thermo Fisher Scientific, Waltham, MA, USA) and incubating overnight at 37 °C. Following overnight digestion, a second aliquot of 100 ng trypsin was added, and the mixture incubated for an additional 2 h at room temperature. The reaction was quenched by the addition of formic acid (Sigma-Aldrich, Saint Louis, MO, USA) to a final concentration of 0.5%, prior to mass spectrometry (MS) analysis.

### 4.4. Liquid Chromatography Mass Spectrometry/Mass Spectrometry (LC-MS/MS) Analysis

Liquid chromatography–tandem mass spectrometry (LC-MS/MS) analysis of OSP peptides was conducted using an Ultimate 3000 nano high-performance liquid chromatography (HPLC) system (Thermo Fisher Scientific, Waltham, MA, USA) for chromatographic separation, coupled to an Orbitrap Velos mass spectrometer (Thermo Scientific, Waltham, MA, USA) via a nanoelectrospray ionization interface. Solvent A consisted of water with 0.1% formic acid, while solvent B was acetonitrile with 0.1% formic acid. Proteolytic peptide samples (200 ng) were loaded onto a trapping column (Thermo, Dionex, Pepmap C18) and maintained at 35 °C for desalting prior to gradient elution. Peptide separation was achieved with a 500 × 0.075 mm analytical column (Reprosil C18-AQ, Dr. Maisch, Ammerbuch, Germany) operating at a flow rate of 0.5 µL/min and maintained at 50 °C, with a gradient from 12% to 40% of solvent B. Eluted peptides were directed into the Orbitrap Velos mass spectrometer via the nanoelectrospray source. The mass spectrometer was operated in a data-dependent acquisition mode and captured up to 10 MS/MS spectra of ions with charge states of +2 or higher in the linear ion trap. Concurrently, full MS survey scans were recorded at a nominal resolution of R = 60,000. The total data acquisition time for each analysis was 150 min.

### 4.5. Bioinformatics Analysis

Protein identification was performed using advanced software with a 1% false discovery rate. The dataset was refined by removing contaminants and reverse database hits. Only proteins with at least two unique peptides were considered for further analysis. Quantification was based on label-free quantification (LFQ) intensities, providing a robust method for comparing protein levels across samples.

Database search and label-free quantification of LCMS/MS data was performed with the MaxQuant software package (version 2.5.5.0). Database search was performed against the proteome of Oryctolagus cuniculus obtained from UniProt (UP000001811, 41,465 entries, downloaded on 26 January 2024). All samples were treated as independent experiments without fractions. Default parameters for database search (FDR for peptide and protein matches at 1%) and label-free quantification were used throughout with the following exceptions: Label-free quantification: LFQ, Re-quantify: true; Match between runs: true.

Three different samples were analyzed: two tissues and one tissue mixture. All were performed in three separate runs to ensure reproducible data (Table 5).

Gene Ontology (GO) analysis was conducted to find functional components across all three branches of the GO hierarchy. The R package (version 2.22.0) “biomaRt” [10] was utilized with the Ensembl dataset “ocuniculus_gene_ensembl” to map proteins to GO terms. Due to the limited number of proteins with annotations retrieved using biomaRt, additional GO annotations were obtained through the UniProt web service R package (version 2.22.0) for pathway analysis based on protein-to-gene mapping. To perform the actual enrichment analysis we used the R package topGO (version 2.54.0) with the following parameters: algorithm = “elim” and statistic = “fisher” [74].

Functional components were identified through an over-representation analysis approach using the KEGG pathway database, as well as other pathway repositories. To determine enriched pathways, the R packages “biomaRt,” “KEGG.db,” and “KEGGREST” were employed for protein and pathway mapping via over-representation analysis [11].

For organ-specific GO term analysis, each sample from the different tissues and organs was analyzed separately. Tissue-specific analyses were performed to identify proteins uniquely present in a given tissue. In addition to analyses based on protein identifiers, quantitative data analysis was also conducted using Label-Free Quantification (LFQ) intensities. Proteins included in the analysis were those quantified in at least 50% of their respective samples and in at least 33% of the other samples. MA plots for all OSPs were generated using logarithmically transformed quantification values. Additionally, various heatmaps were created that displayed the top 50 proteins with the most significant differences between a given tissue and the others.

For comparative statistics, we compared samples from one tissue to the others. The data distribution could not be assessed due to the low number of samples. *p*-values were therefore calculated using the non-parametric Wilcoxon test. To calculate the 5% and 95% confidence intervals for the folds, we applied 1000 replications of a resampling (with replacement). Due to the exploratory nature of the study, a multiple testing correction was not performed.

The MS proteomics data was deposited in the ProteomeXchange Consortium via the PRIDE [75] partner repository (dataset identifiers PXD051701 and 10.6019/PXD051701).

### 4.6. Identification of Proteins/Genes with Uniprot, g:Profiler, and the Human Protein Atlas

The web-based tools UniProt and g:Profiler were utilized to gain further insights into organ-specific genes and secreted proteins identified in the samples. Gene mapping was performed on the MS data using UniProt, after which the mapped genes were converted to their human orthologs via the g:Profiler engine. These genes were then categorized based on their tissue specificity, utilizing the Human Protein Atlas (HPA) database as the reference source. Organ-specific genes and proteins were extracted and classified according to their respective HPA annotations.

### 4.7. Neural Stem Cell Isolation, Culture, and Characterization by Immunostaining

NSCs were isolated from the subventricular zone of 14-day-old rat embryos (isolation was approved by the animal care committee of Heidelberg University and the government of Baden-Württemberg, Germany (G-211/15). The isolated tissue was washed with PBS and then incubated with 0.05% trypsin/EDTA and 0.2% DNase for 5–6 min at 37 °C. The enzyme activity was stopped by adding 10% fetal bovine serum (FBS) and careful pipetting to achieve a single-cell suspension. The cells were then centrifuged for 6 min at 1200 rpm and expanded by plating onto Poly-Ornithine/Laminin-coated plates and growing in medium composed of Dulbecco’s Modified Eagle Medium/F-12 (DMEM/F12) supplemented with L-glutamine, 2.438 g/L sodium bicarbonate, 1% (*v*/*v*) N-2 supplement, 20 ng/mL Epidermal Growth Factor (EGF), 20 ng/mL fibroblast growth factor (FGF), and 1% (*v*/*v*) Penicillin/Streptomycin (P/S). On day 0 of the experiment, one µg/mL of ultrafiltrate (CNS, Liver, OM) was added to the medium. The medium was changed every two days, and the experiment was stopped on day 8 after introducing ultrafiltrate. The cells were then characterized by immunostaining. NSC groups treated with ultrafiltrates (Control, CNS, Liver, and OM) were stained with microtubule-associated protein 2 (MAP2). On day 8 after treatment, NSCs were fixed with 4% (*v/v*) paraformaldehyde (PFA) for 20 min at room temperature (RT). After washing with DPBS, cells were incubated with 0.1% (*v*/*v*) Triton X-100 for 30 min at RT and then incubated with 10% (*v*/*v*) goat serum for one hour at RT. Subsequently, MAP2 primary antibody (Synaptic System #188006) diluted 1:1000 in 10% (*v*/*v*) goat serum was added to the cells and incubated overnight at 4 °C. The next day, cells were washed with DPBS and incubated with 10% (*v*/*v*) goat serum containing a 1:1000 dilution of goat anti-chicken IgY (H+L) secondary antibodies (ThermoFisher #A21449) for one h at RT. The cells were then washed and incubated with 1:10,000 Hoechst 33,342 for 20 min at RT, washed and analyzed with a Zeiss LSM700 Confocal microscope (Carl Zeiss, Mumbai, India).

### 4.8. Neurite Outgrowth Assay

Three random images were captured from each well of a 24-well plate, with triplicate wells for each treatment. The neurite’s length was analyzed using ImageJ software, Java version 1.8.0_345 (64 bit). Rasband, W.S., ImageJ, National Institutes of Health, Bethesda, MA, USA, https://imagej.net/ij/ (accessed on 18 July 2024). Firstly, the image type, brightness, and contrast were adjusted to allow the neurites to be clearly observable. Then, a region of interest (ROI) was manually selected per cell, and four measurements were taken for each image. In total, 30 different measurements were taken from each group.

### 4.9. Data Analysis

Statistical analysis of neurite length was performed using GraphPad Prism software (version 10.3, www.graphpad.com). Results are shown as the mean ± standard deviation (SD). The distribution of normality was evaluated using the Shapiro–Wilk test. Differences between groups were assessed by one-way ANOVA and considered statistically significant when the *p*-value was <0.05.

## 5. Conclusions

This study presents a detailed, comparative proteomic analysis of OSP100 ultrafiltrates derived from rabbit brain, liver, and OM. It sheds light on the diverse array of proteins, SPs and their potential functional roles in various biological processes. We employed a combination of gentle homogenization, ultrafiltration, and advanced proteomic techniques such as gel electrophoresis, liquid chromatography–mass spectrometry (LC-MS/MS), and bioinformatics analysis to successfully identify and characterize a unique set of proteins that are enriched in specific organ tissues. These proteins play crucial roles in a variety of biological functions, including neuronal development, regulation of growth, immune response, lipid and metal binding, and metabolic processes.

Our findings indicate that the protein networks identified within these 100 kDa NOP ultrafiltrates have significant potential for enhancing biological and therapeutic strategies, particularly in the context of neural outgrowth. The brain-specific extracts in particular showed a marked ability to promote neurite length in NSCs compared to the liver and organ mixture extracts. This observation highlights the capacity of brain-derived proteins to support neural development, suggesting their potential utility in neuroregenerative medicine and related therapies.

A critical aspect of this study was the methodology employed to extract and process the OSPs. Our approach deliberately avoided the use of protease inhibitors or enzyme-based treatments so as to preserve the naturally occurring protein profiles and prevent the introduction of exogenous factors that could alter the results. While this approach aligns with our goal of characterizing the natural spectrum of proteins present within each tissue type, it also introduces certain limitations. For example, the lack of protease inhibitors probably allowed some endogenous proteolysis and changes in protein structure during sample preparation, potentially affecting the size distribution and integrity of the proteins detected. However, careful maintenance of low temperature and control of the flow rate and pressures throughout the extraction and filtration processes likely helped to minimize these effects, thereby providing a more accurate representation of the native protein landscape.

Despite these inherent limitations, the semi-quantitative nature of the MS data obtained in this study provides valuable insights into the composition and abundance of proteins across different tissues. The results highlight the presence of tissue-specific proteins and signaling molecules that could serve as potential targets for developing therapeutic interventions. For instance, specific proteins identified in the liver were significantly enriched in pathways related to metabolism and detoxification, whereas proteins from brain ultrafiltrates were involved in pathways associated with neurodevelopment and neuronal maintenance.

Furthermore, our study illustrates the complexity and diversity of protein networks found within these ultrafiltrates and highlights the potential therapeutic value of SPs and OSPs. Given the increasing interest in biologics and peptide-based therapies, a better understanding of the specific roles of these proteins could pave the way for innovative treatments that target a wide range of conditions, including neurodegenerative diseases, cancer, cardiovascular disorders, and metabolic syndromes. Proteins such as Thymosin Beta-4, with its role in axon regeneration, and N-CAM, which facilitates synaptic connectivity, hold promise for the development of interventions that are able to repair damaged neural circuits. Moreover, the targeting of cytoskeletal dynamics or growth cone guidance could be used to treat diseases characterized by impaired neuronal growth or synaptic dysfunction.

Future studies should focus on validating these findings through more rigorous quantitative analyses and functional assays, both in vitro and in vivo. This should further elucidate the roles of these SPs and their potential impact on cell signaling and tissue repair. Additionally, the integration of proteomic data with genomic and transcriptomic profiles may lead to a more holistic understanding of the biological functions and therapeutic potential of these proteins.

In conclusion, this study underscores the critical importance of SPs and OSPs in the development of novel therapeutic strategies. By providing a detailed analysis of the protein composition within ultrafiltrates, we provide a valuable resource for future research aimed at exploring the therapeutic potential of these bioactive molecules. As the field of regenerative medicine continues to evolve, the unique properties of these proteins could give rise to groundbreaking advances in personalized and targeted treatments, thereby improving patient outcomes for a broad spectrum of diseases.

## Figures and Tables

**Table 1:** Significant KEGG pathways and related proteins found in all experiments.

KEGG Pathway	Proteins
Pathways of neurodegeneration	Vesicle-associated membrane protein-associated protein B/C; Tubulin beta-4A chain; ATP synthase subunit beta, mitochondrial; Neurofilament medium polypeptide; Ubiquitin carboxyl-terminal hydrolase isozyme L1; Polyubiquitin-B; microtubule-associated protein; FUS RNA binding protein; Alpha-synuclein; Tubulin alpha chain; Parkinsonism-associated deglycase; Tubulin beta chain; calcium/calmodulin-dependent protein kinase
Glycolysis/Glucone-ogenesis	Aldo-keto reductase family 1 member A1; Aldose 1-epimerase; phosphopyruvate hydratase; phosphoenolpyruvate carboxykinase (GTP); Phosphoglycerate mutase; Triosephosphate isomerase (TIM)
PPAR signaling pathway	Fatty acid binding protein 7; Fatty acid binding protein 5; Fatty acid binding protein 3; Apolipoprotein A-I; Acyl-CoA-binding protein (ACBP); Hydroxymethylglutaryl-CoA synthase (HMG-CoA synthase); phosphoenolpyruvate carboxykinase (GTP); Fatty acid binding protein 2; Perilipin 2; Sterol carrier protein 2

**Table 2:** Summary of identified proteins from CNS OSP100 with neurite outgrowth potential.

Identified Proteins	Sources for Neuronal Outgrowth
N-CAM	[13,14]
Neuromodulin (GAP-43)	[15,16,17]
Doublecortin (DCX)	[18,19,20]
Thymosin Beta-4	[21,22,23]
Vimentin	[24,25]
Synapsin I	[26,27]
Calretinin	[28,29,30]

**Table 3:** Summary of identified proteins from selected CNS OSP100 KEGG pathways.

KEGG Pathway	Identified Proteins
Neurotrophin signaling pathway	14-3-3 protein epsilon Calcium/calmodulin-dependent protein kinase II alpha Crk-like protein Growth factor receptor-bound protein 2
Axon guidance	Calcium/calmodulin-dependent protein kinase II alpha p21-activated kinase 3 Cofilin-2 Enabled homolog (Mena)
MAPK signaling pathway	Crk-like protein Growth factor receptor-bound protein 2 Microtubule-associated protein tau

**Table 4:** Proteins found exclusively in listed tissue samples.

Sample	Protein
Liver	Ribosome binding protein 1
	Hydroxyacyl-CoA dehydrogenase
	Alpha-lactalbumin (Lactose synthase B protein)
	HCV F-transactivated protein 1
Organ Mixture	Zinc finger FYVE-type containing 1
	Pepsin F (EC 3.4.23.1)
	Gamma-crystallin S (Beta-crystallin S)
	Crystallin gamma N
	Nebulin
	Crystallin beta A4
	Crystallin beta B1
	Lambda-crystallin
CNS/Brain	Myelin expression factor 2
	Serine/threonine-protein kinase PAK 3
	protein-tyrosine-phosphatase (EC 3.1.3.48)
	Small ArfGAP 1
	Formin-binding protein 1-like
	Synaptosome associated protein 91
	Metadherin
	SPG11 vesicle trafficking associated
	RUN and FYVE domain containing 3
	Dynein cytoplasmic 1 intermediate chain 2
	ADP-ribosylation factor
	Ankyrin 2
	Alpha-tubulin N-acetyltransferase 1 (Alpha-TAT1) (TAT)
	Microtubule-associated protein RP/EB family member 2
	ENAH actin regulator
	RAN binding protein 1
	Peptidyl-prolyl cis-trans isomerase (EC 5.2.1.8)
	Amidohydrolase-related domain-containing protein
	Eukaryotic translation initiation factor 3 subunit J (eIF3j)
	Profilin
	Dynein axonemal heavy chain 7
	Microtubule-associated protein RP/EB family member 3
	UBC core domain-containing protein
	Protein kinase domain-containing protein
	Coronin
	VLIG-type G domain-containing protein
	Tubulin beta chain
	SRC kinase signaling inhibitor 1
	Myopalladin
	Pleckstrin homology domain containing A7
	Hydroxymethylglutaryl-CoA synthase (HMG-CoA synthase)

**Table 5:** Summary of all runs for each sample.

Sample	First Independent Production Run	Second Independent Production Run	Third Independent Production Run
CNS	CNS 1J 100	CNS 2J 100	CNS 3J 100
Organ Mixture	OM 7J 100	OM 8J 100	OM 9J 100
Liver	LI 4J 100	LI 5J 100	LI 6J 100

## Data Availability

The MS proteomics data was deposited in the ProteomeXchange Consortium via the PRIDE partner repository (dataset identifiers PXD051701 and 10.6019/PXD051701).

## Supplementary Materials

The following supporting information can be downloaded at https://www.mdpi.com/article/10.3390/ijms26146659/s1.
