# Supplementary material for: Organ-Specific Small Protein Networks in 100 kDa Ultrafiltrates: Functional Analysis and Implications for Neuroregenerative Medicine
Source: Int J Mol Sci. 2025 Jul 11;26(14):6659. doi: 10.3390/ijms26146659 (PMC12294768; doi:10.3390/ijms26146659)
Supplement: Supplementary file 1 [file ijms-26-06659-s001.zip › ijms-3567209-supplementary.pdf]

International Journal of *Molecular Sciences*

MDPI

Supplementary material

Organ-Specific Small Protein Networks in 100 kDa Ultrafiltrates: Functional Analysis and Implications for Neuroregenerative Medicine

Jakub Peter Slivka, Chris Bauer, Tasneem Halhouli, Alexander Younsi, Michelle B. F. Wong, Mike K. S. Chan, Thomas Skutella

RESULTS

Table S1. List of mapped tissue-specific genes (from all samples joint for each organ)

OSPU	Genes
**Liver**	ACAT2, ADAR, AIMP1, AK2, AKR1A1, AP2B1, ATP5F1B, B2M, BIN1, BPHL, CA2, CALD1, CAMK2A, CFL2, CMPK1, COMT, CPS1, CST3, CTSB, CTTN, CYB5A, DBI, DDAH1, DSTN, EEF1A1, EEF2, EIF4H, ELAVL1, EPB41L1, ETFA, ETFB, FKBP3, FN1, FUS, GALM, GAPDH, GMFB, GOT2, GRB2, GSTO1, GSTZ1, H1-3, HADH, HDLBP, HINT1, HMGCS2, HNRNPAB, HNRNPC, HNRNPD, HNRNPH1, HNRNPK, HNRNPL, HNRNPU, HPD, HSPD1, ILF3, KRT8, LASP1, LYPLA1, MAP4, MARCKSL1, MDH2, MIF, MT1A, MTPN, NCL, NPC2, NSFL1C, NXF1, PARK7, PCBP1, PCBP2, PCK2, PCMT1, PCNP, PDIA3, PDIA6, PEBP1, PFN1, PHPT1, PLIN2, PPIB, PPP1R8, PRDX6, PSAP, PSIP1, RAB10, RAB11A, RAB14, RAB2A, RAB7A, RAD23B, RGN, RRBP1, S100A11, SCP2, SELENBP1, SERBP1, SF1, SF3B2, SFPQ, SH3BGRL, SNX12, SRSF3, SUB1, TBCB, THRAP3, TST, TUBA4A, TUBB, TUBB6, TXN, TXNL1, UBB, VAPB, VIL1, VIM, YWHAE, YWHAG, YWHAZ
**OM**	EEF2, ABRACL, DBI, SH3BGRL3, S100A11, PFN1, TXN, SNCA, MIF, PHPT1, HINT1, EFHD2, PEA15, DSTN, FABP3, LGALS1, CYB5A, GMFB, LYZ, NPC2, PTGES3, SRSF3, TPT1, PCNP, CRYGS, PDCD5, HNRNPAB, TAGLN2, DDAH1, MTPN, PEBP1, MARCKSL1, PARK7, RHOA, CFL2, ARHGDIB, SH3BGRL, RAB7A, RAB2A, RAB14, PPIB, RAB11A, GSTZ1, H1-1, H1-3, UCHL1, PRDX6, FKBP3, CMPK1, RAB10, AK2, CLIC1, GSTO1, ETFB, TBCB, YWHAG, YWHAZ, SNX12, VAPB, CA2, AGR2, STX7, HEBP1, COMT, MAPRE1, LYPLA1, HNRNPD, PCMT1, UBB, TXNL1, BPHL, RGN, YWHAE, HNRNPC, TST, ETFA, MDH2, MARCKS, AIMP1, PCBP2, CTSB, GALM, PCBP1, AKR1B10, EIF4H, CRYL1, NSFL1C, LASP1, ACTB, ELAVL1, ACAT2, RAD23B, AKR1A1, DDX39B, HNRNPH1, GOT2, NXF1, PDIA6, TUBA4A, HPD, IDH2, EEF1A1, HNRNPK, VIM, CAMK2A, KRT8, SELENBP1, PLIN2, PDIA3, PSAP, FUS, ATP5F1B, PSIP1, TUBB6, HMGCS2, CALD1, SFPQ, SCP2, EHD1, BIN1, PPP1R8, HSPD1, WDR1, ALB, DBN1, SF1, HSPA2, PCK2, PDLIM5, ZFYVE1, DDX17, NCL, HNRNPL, ACO2, HNRNPU, VIL1, MAPT, SF3B2, ILF3, THRAP3, AP2B1, ADAR, HDLBP, EPRS1, MAP2, FN1, SPTAN1, CSTB, FABP5, EIF5A, AK1, TAGLN3, CA1, DCLK1, ACTA1, ENO2, LDB3, NEFH, NCAM1, FABP2, RBP2, FABP7, CPS1, CRABP2, AKR1B1, CRYGD, CRYGA, CRYBA4, CRYBA2, CRYBA1, CRYBB3, CRYBB1
**Brain**	EEF2, ABRACL, DBI, PFN2, SH3BGRL3, S100A11, PFN1, TXN, SNCA, MIF, B2M, PHPT1, HINT1, EFHD2, PEA15, DSTN, LGALS1, GMFB, CST3, EIF5A, JPT1, FABP7, PTGES3, SRSF3, TRIR, PCNP, PDCD5, HNRNPAB, DDAH1, TAGLN2, MTPN, PEBP1, PARK7, MARCKSL1, AK1, CFL2, TAGLN3, SH3BGRL, RAB7A, EIF3J, SUB1, RAB2A, RAB14, RAB11A, PPIB, GRB2, H1-3, UCHL1, FKBP3, PRDX6, CMPK1, RAB10, AK2, GSTO1, TBCB, YWHAG, YWHAZ, SNX12, VAPB, CA2, STX7, HEBP1, COMT, MAPRE1, CALB2, LYPLA1, HNRNPD, PCMT1, UBB, TXNL1, YWHAE, HNRNPC, TST, MDH2, MAPRE2, ATAT1, GAPDH, MARCKS, AIMP1, PCBP2, CTSB, PCBP1, DCX, DCLK1, EIF4H, NSFL1C, LASP1, ACTB, TMOD2, ELAVL1, SERBP1, RAD23B, HNRNPH1, GOT2, ENO2, SMAP1, NXF1, PDIA6, TUBB, TUBB4A, TUBA4A, IDH2, EEF1A1, HNRNPK, VIM, CAMK2A, ENAH, PDIA3, TUBB3, PSAP, FUS, CORO1C, ATP5F1B, PSIP1, CELF2, CALD1, TUBB6, CTTN, HMGCS1, SFPQ, EHD1, BIN1, PPP1R8, HSPD1, WDR1, MYEF2, FNBP1L, DYNC1I2, RUFY3, DBN1, SF1, NEFM, DPYSL3, SYN1, AMPH, DCLK2, DDX17, NCL, HNRNPL, EPB41L1, ACO2, HNRNPU, MAPT, SF3B2, ILF3, SNAP91, THRAP3, AP2B1, NEFH, ADAR, RTN4, NCAM1, PLEKHA7, HDLBP, MAP4, SRCIN1, EPRS1, MAP2, PTPRZ1, SPG11, SPTAN1, ANK2, FABP5, BPHL, AKR1B1, PAK3

International Journal of Molecular Sciences

MDPI

Table S2. Overview of the tissue specific GO:terms analysis

OSPU	GO:terms
Liver	GO:0010604: positive regulation of macromolecule metabolic process<br>GO:1902936: phosphatidylinositol bisphosphate binding<br>GO:0072673: lamellipodium morphogenesis<br>GO:1903900: regulation of viral life cycle<br>GO:0030855: epithelial cell differentiation<br>GO:0070161: anchoring junction<br>GO:0140694: non-membrane-bounded organelle assembly<br>GO:0060627: regulation of vesicle-mediated transport<br>GO:0010629: negative regulation of gene expression<br>GO:0030029: actin filament-based process<br>GO:0007163: establishment or maintenance of cell polarity<br>GO:0030833: regulation of actin filament polymerization<br>GO:0043226: organelle<br>GO:0035728: response to hepatocyte growth factor<br>GO:0035729: cellular response to hepatocyte growth factor stimulus<br>GO:0051640: organelle localization<br>GO:0032530: regulation of microvillus organization<br>GO:0055002: striated muscle cell development<br>GO:0009892: negative regulation of metabolic process<br>GO:0008064: regulation of actin polymerization or depolymerization<br>GO:0030832: regulation of actin filament length<br>GO:0048638: regulation of developmental growth<br>GO:0031647: regulation of protein stability<br>GO:0043489: RNA stabilization<br>GO:1902373: negative regulation of mRNA catabolic process<br>GO:1902369: negative regulation of RNA catabolic process<br>GO:0034250: positive regulation of cellular amide metabolic process<br>GO:1901881: positive regulation of protein depolymerization
Organ mix	GO:0051346: negative regulation of hydrolase activity<br>GO:0071826: ribonucleoprotein complex subunit organization<br>GO:0070508: cholesterol import<br>GO:0033700: phospholipid efflux<br>GO:0019915: lipid storage<br>GO:0048523: negative regulation of cellular process
Brain	GO:0015031: protein transport<br>GO:0031400: negative regulation of protein modification process<br>GO:0051641: cellular localization

	GO:0006469: negative regulation of protein kinase activity<br>GO:0009890: negative regulation of biosynthetic process<br>GO:0010558: negative recgulation of macromolecule biosynthetic process<br>GO:0042995: cell projection<br>GO:0031327: negative regulation of cellular biosynthetic process<br>GO:0006996: organelle organization<br>GO:0031982: vesicle<br>GO:0005496: steroid binding<br>GO:0070887: cellular response to chemical stimulus<br>GO:0034330: cell junction organization<br>GO:0034097: response to cytokine

International Journal of
Molecular Sciences

MDPI

Table S3. List of all small proteins found. Listed alphabetically.

10 kDa heat shock protein, mitochondrial (10 kDa chaperonin) (Chaperonin 10)
14 kDa phosphohistidine phosphatase (EC 3.9.1.3) (Phosphohistidine phosphatase 1) (PHPT1) (Protein histidine phosphatase) (PHP)
Acyl-CoA-binding protein (ACBP) (Diazepam-binding inhibitor) (DBI) (Endozepine) (EP)
Acylphosphatase (EC 3.6.1.7)
Adenosine 5'-monophosphoramidase HINT1 (EC 3.9.1.-) (Desumoylating isopeptidase HINT1) (EC 3.4.22.-) (Histidine triad nucleotide-binding protein 1) (P13.7)
Alpha-lactalbumin (Lactose synthase B protein)
Alpha-synuclein
Antioxidant 1 copper chaperone
Astrocytic phosphoprotein PEA-15 (15 kDa phosphoprotein enriched in astrocytes)
Beta-2-microglobulin
BolA family member 2
Calmodulin (CaM)
Cellular retinoic acid binding protein 2
Costars family protein ABRACL (ABRA C-terminal-like protein)
Cystatin B
Cystatin-C (Cystatin-3)
Cytochrome b5
Cytochrome c domain-containing protein
Destrin, actin depolymerizing factor
Dynein light chain 1, cytoplasmic (8 kDa dynein light chain) (DLC8) (Dynein light chain LC8-type 1) (Protein inhibitor of neuronal nitric oxide synthase) (PIN)
EF-hand domain family member D2
Elongation factor 2 (EF-2)
Enhancer of rudimentary homolog
Eukaryotic translation initiation factor 5A (eIF-5A)
Fatty acid binding protein 2
Fatty acid binding protein 3
Fatty acid binding protein 5
Fatty acid-binding protein, liver (L-FABP) (Liver-type fatty acid-binding protein)
Galectin
Glia maturation factor
Glutaredoxin-1
GTP-binding nuclear protein Ran
HCV F-transactivated protein 1
Hemoglobin subunit alpha-1/2 (Alpha-1/2-globin) (Hemoglobin alpha-1/2 chain)
Hemoglobin subunit beta-1/2 (Beta-1/2-globin) (Hemoglobin beta-1/2 chain)
Hemoglobin subunit gamma (Gamma-globin) (Hemoglobin beta-3) (Hemoglobin gamma chain)
Jupiter microtubule associated homolog 1
Lysozyme (Lysozyme F1)
Macrophage migration inhibitory factor
Mammalian defensins domain-containing protein

Metallothionein-1A (MT-1A) (Metallothionein-IA) (MT-IA)
Metallothionein-2A (MT-2A) (Metallothionein-IIA) (MT-IIA)
Metallothionein-2D (MT-2D) (Metallothionein-IID) (MT-IID)
Morphine 6-dehydrogenase (EC 1.1.1.218)
Myelin P2 protein
Myoglobin
Nuclear transport factor 2 (NTF-2)
Peptidyl-prolyl cis-trans isomerase FKBP1A (PPIase FKBP1A) (EC 5.2.1.8) (12 kDa FK506-binding protein) (12 kDa FKBP) (FKBP-12) (Calstabin-1) (FK506-binding protein 1A) (FKBP-1A) (Immunophilin FKBP12) (Rotamase)
Profilin
Protein S100-A11 (Calgizzarin) (Protein S100-C) (S100 calcium-binding protein A11)
Prothymosin alpha
Retinol binding protein 2
SH3 domain-binding glutamic acid-rich-like protein
Small ubiquitin-related modifier (SUMO)
Stathmin
Thioredoxin (Trx)
Thioredoxin domain-containing protein 17
Thymosin beta
Thymosin beta-4 (T beta 4) [Cleaved into: Hematopoietic system regulatory peptide (Seraspenide)]
UBC core domain-containing protein
Uncharacterized protein
Whey acidic protein (WAP)

International Journal of
Molecular Sciences

MDPI

DISCUSSION

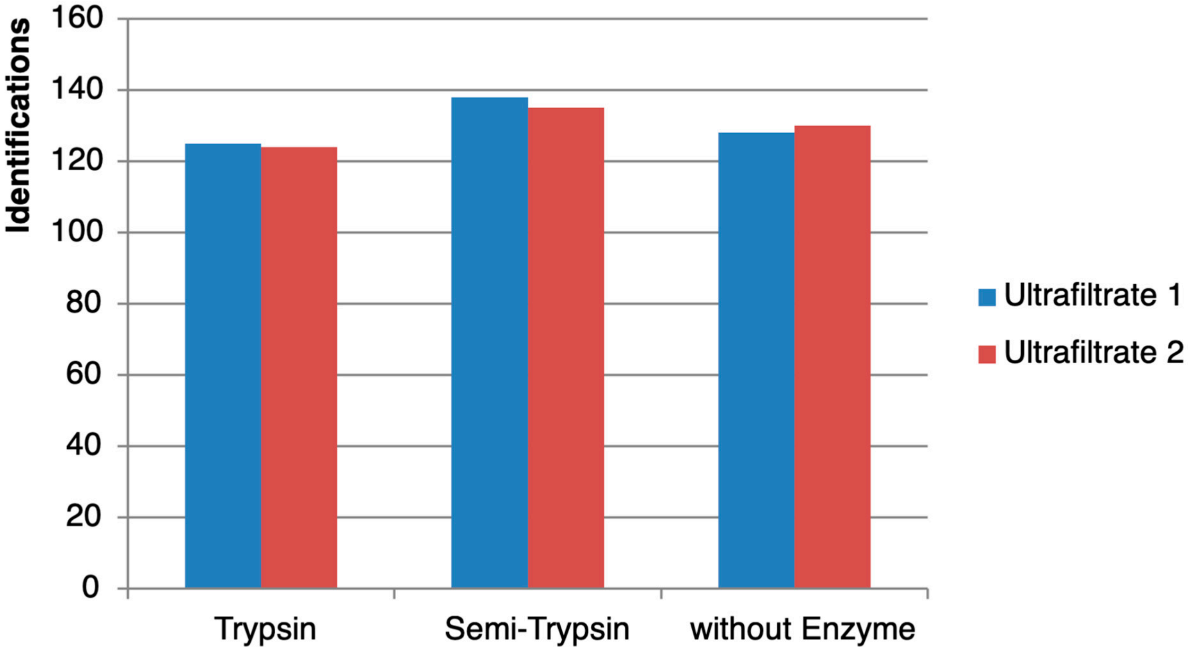

FIGURE S1. Analysis of potential protein degradation in ultrafiltrated samples. Two exemplary measurement data sets (Ultrafiltrate 1 and 2) were each analyzed three times using different MASCOT search parameters:

1. Standard analysis: Protein digestion with trypsin.
2. Semi-trypsin analysis: Searches for peptides where one cleavage site is specific for trypsin, while the other cleavage site can be non-specific (e.g., N- or C-terminal ends may also be detected).
3. Analysis without a specific enzyme: Simulates a non-specific digestion, where a higher number of protein identifications would be expected compared to digestion with a defined protease.

The number of identified proteins was only slightly increased under non-specific conditions, suggesting that only minor degradation occurred.
